# Mixture-modeling approach reveals global and local processes in visual crowding

**DOI:** 10.1038/s41598-022-10685-z

**Published:** 2022-04-25

**Authors:** Mikel Jimenez, Ruth Kimchi, Amit Yashar

**Affiliations:** 1 Department of Special Education, Faculty of Education, University of Haifa, 199 Abba Khoushy Ave, Haifa, 3498838 Israel; 2 Department of Psychology, School of Psychological Sciences, University of Haifa, Haifa, Israel; 3 Institute of Information Processing and Decision Making, University of Haifa, Haifa, Israel; 4 The Edmond J. Safra Brain Research Center for the Study of Learning Disabilities, The University of Haifa, Haifa, Israel

**Keywords:** Object vision, Human behaviour

## Abstract

Crowding refers to the inability to recognize objects in clutter, setting a fundamental limit on various perceptual tasks such as reading and facial recognition. While prevailing models suggest that crowding is a unitary phenomenon occurring at an early level of processing, recent studies have shown that crowding might also occur at higher levels of representation. Here we investigated whether local and global crowding interference co-occurs within the same display. To do so, we tested the distinctive contribution of local flanker features and global configurations of the flankers on the pattern of crowding errors. Observers (n = 27) estimated the orientation of a target when presented alone or surrounded by flankers. Flankers were grouped into a global configuration, forming an illusory rectangle when aligned or a rectangular configuration when misaligned. We analyzed the error distributions by fitting probabilistic mixture models. Results showed that participants often misreported the orientation of a flanker instead of that of the target. Interestingly, in some trials the orientation of the global configuration was misreported. These results suggest that crowding occurs simultaneously across multiple levels of visual processing and crucially depends on the spatial configuration of the stimulus. Our results pose a challenge to models of crowding with an early single pooling stage and might be better explained by models which incorporate the possibility of multilevel crowding and account for complex target-flanker interactions.

## Introduction

Recognition of objects is limited by their spacing. When objects are too close together, they become indistinguishable, a phenomenon known as crowding. Crowding sets a fundamental limit on conscious visual perception^1^ and impairs reading, eye and hand movements, visual search and other functions in typical peripheral and central amblyopic vision^2^.

The study of crowding has gained increased attention in recent years, and our knowledge of crowding characteristics is substantial^1,3–5^. For example, crowding impairs target identification but not detection^6,7^ and it depends on both target eccentricity and the distance between target and flankers (i.e., the critical spacing, 0.3–0.5 of target eccentricity)^7–10^ (but see^11,12^). In addition, crowding depends on the similarity between the target and the flankers, such that flankers more similar to the target (e.g., in color, shape, depth, spatial frequency or complexity) produce stronger crowding^13–15^.

Nonetheless, the underlying processes by which crowding occurs are still unclear. Most of the prevailing theories of crowding propose that crowding occurs because of the integration or ‘‘pooling’’ of low-level features at a single, relatively early stage of visual processing^7,16,17^, in line with the classic hierarchical model of object recognition^18–20^. In these models, information proceeds from the processing of low-level visual features (such as spatial frequency, orientation or color) to the perception of coherent forms, shapes and complex objects. Importantly, the input of higher-level visual areas would be fully determined by the outputs of basic, lower-level feature detectors, and thus information lost at early stages of visual processing would be irretrievably lost^21,22^. If crowding occurs due to the averaging of low-level visual features between target and flankers at an early visual stage, it would disrupt the formation of any higher-level target representation, and crowding would represent an early bottleneck of human vision.

However, in contrast to the prediction of a strict “pooling” or averaging mechanism, crowding errors often reflect reports of a flanker instead of the target—known as misreport errors^23–27^. Interestingly, misreport errors lead to feature-binding errors—i.e., reporting the color of the target along with the orientation of a flanker^27^, indicating that crowding reflects a more complex integration failure than a simple averaging.

Classic models of crowding are also challenged by recent findings showing that crowding can occur at different levels of visual processing, not only between lower-level features but also between high-level representations of objects. Indeed, crowding may occur for complex stimuli like shapes^28,29^, everyday objects^30^ or faces^31–33^ (see Levi^3^ for a review). A particular line of research has focused on how perceptual grouping processes and configurational effects modulate crowding^6,34,35^. Within this line, Kimchi and Pirkner^28^ examined whether crowding could occur at the object configural level in addition to feature- or part-level crowding. Their results showed that crowding was weaker when the flankers were similar to the target’s local parts than when the flankers were similar to the target’s global configuration. Herzog et al.^22^ found that the same vertical flankers lose their crowding strength when becoming part of rectangles or good Gestalts, suggesting that the high-level feature processing might interfere with low-level feature processing. Results by Francis, Manassi and Herzog^36^ suggest that when flankers form a large group and the target is not connected, crowding is released by segmentation processes. More recently, Doerig, Bornet, Rosenholtz, Francis, Clarke and Herzog^37^ showed that crowding models that incorporate a grouping component strongly improve model performance, as crowding seem to depend not only on whether the flankers are part of a larger Gestalt percept, but more generally on the configuration of flankers in the whole visual field^38^.

Overall, research on configurational effects on crowding have thoroughly explored how crowding strength varies when flankers group together with the target or, alternatively, form a separated group^22,37,38^. In the former case, crowding is strong, in the latter, crowding is weak: the target is released from crowding because the flankers are processed as a different group. These findings suggest that perceptual grouping processes between target and flankers play a crucial role in crowding interference. However, it is still unknown how crowding is affected by different grouping processes and the specific pattern by which crowding operates at the global and local levels of stimulus representation.

**Figure 1:**
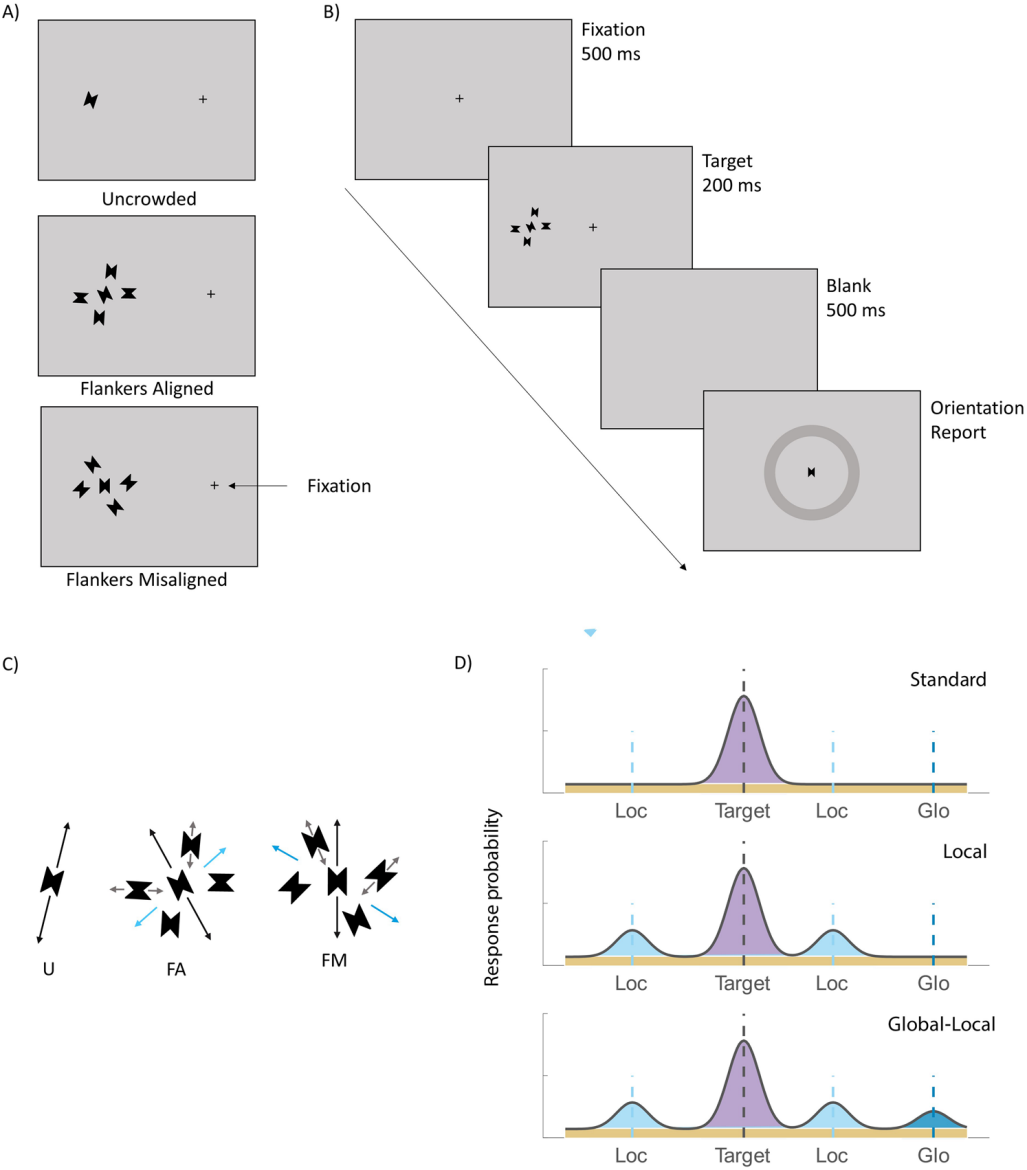
Stimulus type and display conditions. **(A)** The three display conditions. From top to bottom, *U* uncrowded, *FA* flankers aligned, *FM* flankers misaligned. **(B)** Illustration of the sequence of events within a trial. **(C)** Depiction of target orientation (black arrow), local flanker orientations (grey arrows) and global shape orientations (light and dark blue arrows) in flankers aligned and flankers misaligned conditions, respectively. **(D)** Three models of response distribution, illustrated for a single crowded display trial. The standard model (top) is a mixture of a Gaussian distribution over the target orientation, which represents the variability of target reports, and a uniform distribution, which represents guesses. The Local model (middle) adds to the standard model a component of misreporting the local flankers as the target. The Global–Local model adds to the Local model a component of misreporting the global orientation as the target.

In this study, we introduce a mixture modeling approach to address these questions by exploring whether and how crowding errors (i.e., the pattern of misreport errors between target and flankers) depend on the level of visual processing. Observers had to report the orientation of a target, a black rectangle with two triangular cut-outs, which was either presented alone or surrounded by flankers in two different conditions (see Fig. 1A). In both crowding conditions the target was flanked by four similar flankers, each placed at a corner of an imaginary rectangle. In the flankers-aligned condition, the flankers were aligned to create a coherent illusory rectangular shape. Illusory—or Kanizsa—shapes^39^ refer to the perception of a shape defined by sharp illusory contours (see^40^ for a review). In the flanker-misaligned condition, no illusory shape was obtained, but flankers might group together to form a global rectangular configuration. In both conditions, the global configuration, either the illusory rectangle or the rectangular configuration, had an orientation (global orientation) that varied independently from the orientation of the individual flankers (local orientation), and from the target’s orientation (see Fig. 1C). Note however, that the two conditions varied in the strength (or goodness) of the global configuration and, not unrelated, the strength of the grouping of the flankers. Both the global configuration and the grouping of the flankers were presumably stronger in the flankers aligned condition, which was governed by Gestalt principles such as collinearity and closure (in addition to proximity and similarity that were present also in the flankers misaligned condition).

For each trial, we calculated the estimation error for orientation by subtracting the true value of the target from the estimation value and analyzed the error distributions by fitting probabilistic mixture models. If crowding occurs only at a lower level of visual processing, we expected crowding to be induced by local shapes, but not by global configuration. Namely, observers would only misreport the orientation of a flanker, but not the orientation of the global shape formed by the flankers, as the target orientation. If both low-level (individual flankers) and high-level (global configuration) stimulus representations are preserved and crowding occurs also at a higher level of visual processing, we expected crowding to be induced by local shapes, but also by global configurations. That is, observers would misreport local as well as global orientations as the target orientation. Manipulating the strength of the global configuration and the grouping of the flankers would allow us to examine how different perceptual organization processes modulate crowding.

## Methods

### Observers

Twenty-seven graduate and undergraduate students (17 females: age range = 18–39 years, *M* = 28.23, *SD* = 6.07) from The University of Haifa participated in the experiment for either course credit or monetary payment (40 ILS per hour, around $12). The sample size was calculated on the basis of an a priori power analysis to detect a crowding effect with 80% power and a moderate effect size (0.5), given a 0.05 significance criterion. All observers had normal or corrected-to-normal visual acuity and normal color vision. None of them reported either attention deficits or epilepsy. A written informed consent was signed by the participants before the experiment. The method was carried out in accordance with the Declaration of Helsinki and was approved by the Human Ethics Committee of the University of Haifa.

### Apparatus

The stimuli were displayed on a gamma-corrected 21-in CRT monitor (SGI, with 1280 × 960 resolution and 85-Hz refresh rate) connected to an iMac and were programmed in Matlab (The MathWorks, Inc., Natick, MA) using the Psychophysics Toolbox extensions^41^. An Eyelink 1000 Plus (SR Research, Ottawa, ON, Canada) system was used to monitor eye movements, and viewing distance was set to 57 cm using a chin rest. Observers used the mouse to report their responses.

### Stimuli

The stimuli were presented on a grey background with a luminance level of 56 cd/m^2^. The fixation display consisted of black cross subtending 0.3° at the center of the screen. The target display consisted of the fixation cross along with the target; a rectangular black (0 cd/m^2^) shape subtending 1.1° in height and 0.9° in width, presented on the horizontal meridian, either on the left or on the right hemifield, and with 9° eccentricity. A triangular shape (0.1°) was cut out from the two sides of the rectangle (Fig. 1A). The target could appear alone (uncrowded condition) or surrounded by four flankers in two different flanker configurations: flankers aligned and flankers misaligned conditions. The flanker stimuli were identical to the target stimulus but of different orientation. The center-to-center target-flanker spacing was 1.8º.

Target and flanker orientations were selected randomly from a circular parameter space, which consisted of 180 values evenly distributed between 1° and 180°. In both the flankers aligned and flankers misaligned conditions, diagonal flankers had always the same orientation, i.e., flankers were always rotated in pairs. Thus, the number of unique flanker orientations in each flanker condition was always two. In the flankers aligned condition, flankers’ triangular corners were aligned so they created an illusory rectangle (2° in height and 3° in width, see Fig. 1A). In the flankers misaligned condition, the pair of flankers were rotated randomly in each trial.

### Procedure and design

Figure 1B illustrates the trial sequence. We instructed observers to fixate on the fixation cross during the trial presentations. Each trial began with the presentation of the fixation display for 500 ms, which continued until the observer fixated consecutively on the fixation cross for 300 ms. Following observer’s fixation, the target display appeared for 200 ms. After the stimulus display, a blank screen was presented for 500 ms, which was followed by a response screen. During the response display, observers estimated the target orientation by selecting a position for the target stimulus on an orientation wheel (see Fig. 1B). Following the observer’s response, a blank inter-trial interval (ITI) appeared for 500 ms.

Observers were presented with 200 trials for each of the three display conditions (uncrowded, flankers aligned and flankers misaligned), for a total of 600 trials per session. The experiment was divided in 10 blocks of 60 trials and lasted approximately 60 min. Display conditions were randomly presented within each block. Observers were advised to take short rests between blocks.

In each trial, eye fixation was monitored using an eye tracker (see “Apparatus”) and a fixation window of 2° in diameter (fixation window was increased to 3° for seven subjects who had difficulties fixating within the 2° fixation window). Trials in which fixation was broken were eliminated from the data and rerun at the end of the block.

### Models and analyses

The estimation error for orientation reports was calculated by subtracting the true value of the target from the estimation value at each trial, such that zero indicated the target value. We calculated flankers’ values by subtracting the true value of the target from the values of the flankers in order to assess the contribution of the flankers to the error distribution. The error distributions were analyzed by fitting probabilistic mixture models, developed from both the standard model and the standard with misreport model^42^. We compared three different models:

The Standard Mixture model (see Fig. 1D) with two components: a von Mises (circular) distribution that describes the probability density of reports around the target’s orientation, and a uniform distribution that describes the probability of reports that are unrelated to the target (guessing rate). In this model, the probability of reporting a feature value $p\left(\widehat{\theta }\right)$ is

$$p\left(\widehat{\theta }\right)=\left(1-\gamma \right){\phi }_{\sigma }\left(\widehat{\theta }-\theta \right)+\gamma \left(\frac{1}{180}\right)$$

where $\widehat{\theta }$ is the reported orientation and $\theta$ is the target orientation, ${\phi }_{\sigma }$ is the von Mises distribution with mean of 0 and standard deviation $\sigma$ (variability), and $\gamma$ is the proportion of trials on which observers reported at random (guessing rate). Thus, the model includes two free parameters (γ, σ).

The Local model adds a misreporting component to the standard mixture model, which describes the probability of reporting the orientation of any of the four local flankers to be the target. In this model, the probability of reporting a feature value $p\left(\widehat{\theta }\right)$ is

$$p\left(\widehat{\theta }\right)=\left(1-\gamma -{\beta }^{Loc}\right){\phi }_{\sigma }\left(\widehat{\theta }-\theta \right)+\gamma \left(\frac{1}{180}\right)+{\beta }^{Loc}\frac{1}{m}\sum_{i}^{m}{\phi }_{\sigma }\left(\widehat{\theta }-{\varphi }_{i}^{Loc}\right)$$

where ${\beta }^{Loc}$ is the probability of reporting a flanker orientation as the target orientation, $m$ represents the total number of nontarget items ($m=4$ in the present study), and ${\varphi }_{i}^{Loc}$ is the orientation of the $i$th flanker. The model has three free parameters.

Finally, the Global–Local adds a global configuration misreporting component to the local model, which describe the probability of reporting the orientation of the global configuration of the four flankers (see Fig. 1C). In this model, the probability of reporting a feature value $p\left(\widehat{\theta }\right)$ is

$$p\left(\widehat{\theta }\right)=\left(1-\gamma -{\beta }^{Loc}-{\beta }^{Glo}\right){\phi }_{\sigma }\left(\widehat{\theta }-\theta \right)+\gamma \left(\frac{1}{180}\right)\,+\, {\beta }^{Loc}\frac{1}{m}\sum_{i}^{m}{\phi }_{\sigma }\left(\widehat{\theta }-{\varphi }_{i}^{Loc}\right)\,+\, {{\beta }^{Glo}\phi }_{\sigma }\left(\widehat{\theta }-{\varphi }^{Glo}\right)$$

where ${\beta }^{Glo}$ is the probability of reporting the orientation of the global configuration, and ${\varphi }_{i}^{Glo}$ is the orientation of the global configuration.

We used the MemToolbox^43^ to fit and compare the models to the individual data. For model comparison, we calculated the Akaike information criterion with correction (AICc) to the individual fits. We then performed statistical testing to the individual AICcs and to the parameters of the individual fits.

## Results

**Figure 2:**
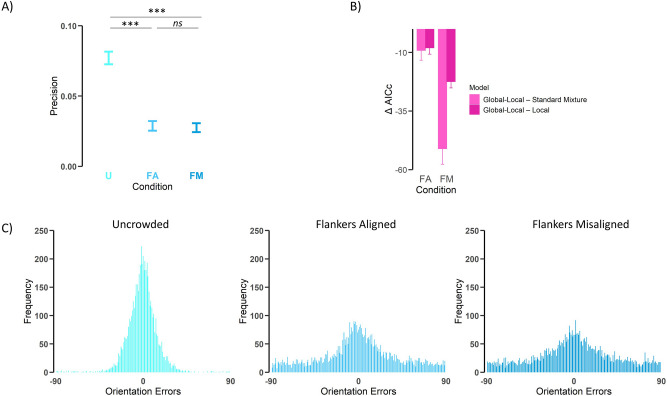
Error distributions, model comparison and precision. **(A)** Mean precision (inversed SD in degrees) of the errors in each display condition, *U* uncrowded, *FA* flankers aligned, *FM* flankers misaligned. **(B)** ∆AICc comparisons for each flanker condition. In both conditions the Global–Local model outperformed both the Standard Mixture and the Local models (lower AICc indicates better model fit). ∆AICcs was calculated by subtracting the AICc of both Standard Mixture and Local models from that of the Global–Local model (AICc_Global–local_ – AICc_StandardMixture_, AICc_Global–local_ – AICc_Local_). **(C)** Frequency of errors (reported value minus true value of the target) for each of the three display conditions. Note that because flankers were uniformly distributed compared to the target, misreports rates form a uniform shape in the plot. Error bars represent standard error.

For each observer in each condition, we examined the bias of the errors by calculating the mean error. Mean error was close to zero in the uncrowded condition (*M* = −0.78, *SD* = 1.57), the flankers aligned (*M* = 0.77, *SD* = 3.14) and flankers misaligned (*M* = 1.12, *SD* = 3.68) conditions. We then calculated precision as the inverse of the variance of the errors for each observer in each condition (Fig. 2A). We conducted a one-way Analysis of Variance (ANOVA) on precision with display conditions (uncrowded, flankers aligned, flankers misaligned) as a within subject factor. A main effect on precision was observed, *F*(2,52) = 130.59, *p* < 0.001, *ƞ*_p_^2^ = 0.83, indicating significant differences in precision between the three display conditions. Pairwise comparisons (Bonferroni corrected) showed that precision was significantly higher when the target was presented uncrowded compared to when it was presented surrounded by flankers (aligned: *p* < 0.001; misaligned: *p* < 0.001). On the other hand, no differences in precision were found between the two flanker conditions (*p* = 0.091).

### Probabilistic models

In the uncrowded condition, the standard mixture model described accurately the distribution of the errors (Fig. 2C). For each flanker condition, we compared the three relevant models (e.g., the Standard Mixture model, the Local model and the Global–Local model) by calculating the Akaike information criterion with correction (AICc) for each observer. Figure 2B shows the mean AICc differences between the models in each flanker condition. The Global–Local model outperformed (i.e., lower AICc value) the Standard Mixture model and the Local model in both the flankers aligned and flankers misaligned conditions [Global-Local–Standard Mixture: *t*(26) = 2.17, *p* = 0.039, Cohen’s *d* = 0.42; Global–Local-Local: *t*(26) = 2.98, *p* = 0.006, Cohen’s *d* = 0.57, in the FA condition; Global–Local-Standard Mixture: *t*(26) = 7.70, *p* < 0.001, Cohen’s *d* = 1.48; Global–Local-Local: *t*(26) = 8.30, *p* < 0.001, Cohen’s *d* = 1.59, in the FM condition]. Following these results, we analyzed the fitted parameters of the best performing models (i.e., the Standard Mixture for the uncrowded display, and the Global–Local model for the two types of crowded displays) in each condition.

**Figure 3:**
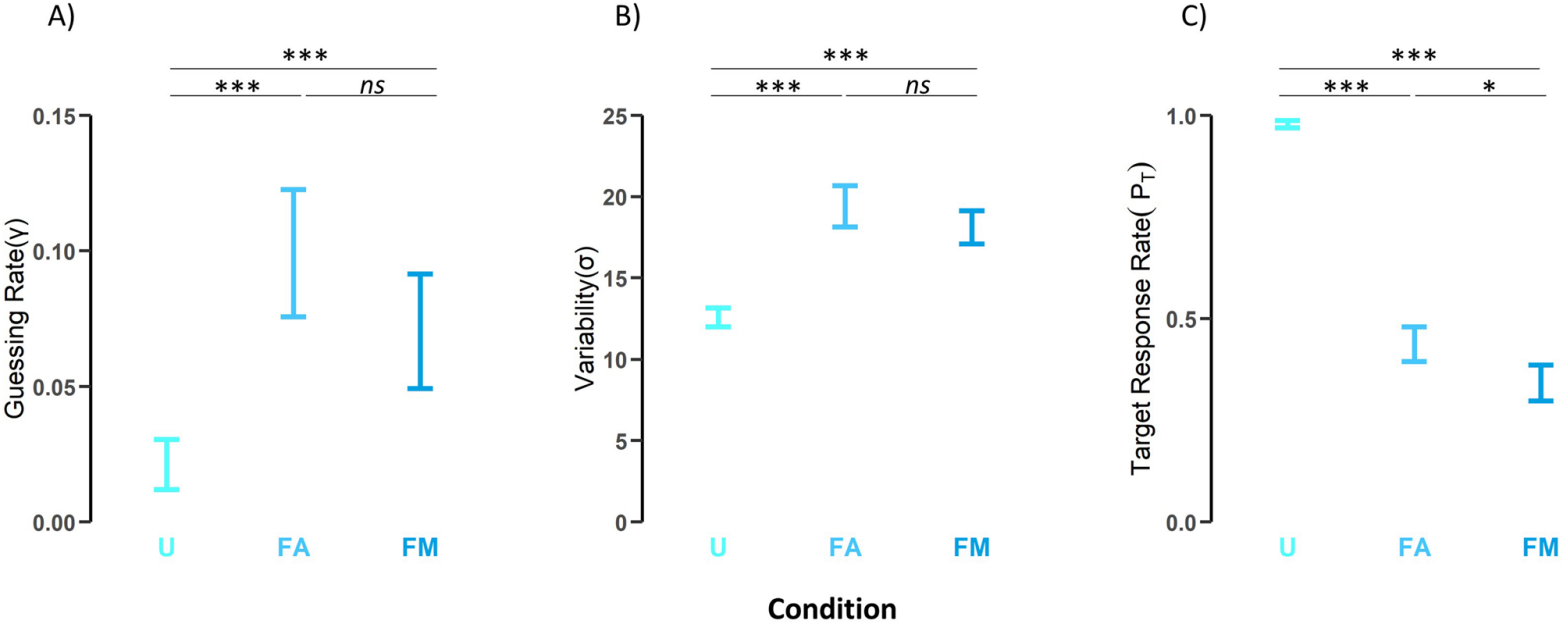
Model parameters from the fitting of the Global–Local model. (**A**) Guessing rate (γ), (**B**) Mean variability (σ, in degrees), and (**C**) target reporting rate (P_*T*_) for each display condition. *U* uncrowded, *FA* flankers aligned, *FM* flankers misaligned. Error bars represent standard error.

We calculated target reporting rate (*P*_*T*_) by subtracting the accumulative guessing rate and misreport rate from 1 (i.e., *P*_*T*_ = 1 – γ for the Standard Mixture, *P*_*T*_ = 1 − γ – β_global_ – β_local_ for the Global–Local model) for each fitted model. Figure 3 depicts the mean guessing rate (γ), variability (σ) and target reporting rate (*P*_*T*_) of the fitted models in each condition. To assess the effect of crowding on performance, we conducted one-way, repeated measure ANOVAs on guessing rate, variability and target reporting rate as dependent variables, with display condition as a within subject factor. For guessing rate, a main effect of display condition [*F*(2,38) = 4.99, *p* = 0.019, *ƞ*_p_^2^ = 0.16] suggested differences between conditions (note that Greenhouse–Geisser corrections are applied on *p*-values and degrees of freedom when the sphericity assumption is violated). Pairwise comparisons showed that the guessing rate was significantly lower when the target was presented alone (uncrowded), than when it was flanked (aligned: *p* = 0.015; misaligned: *p* = 0.024), yet no differences were found between the two flanker conditions (*p* = 1.000). For variability, a main effect of crowding [*F*(2,42) = 34.11, *p* < 0.001, *ƞ*_p_^2^ = 0.57] also suggested differences between conditions. Similar to the pattern for guessing rate, variability in the orientation reports was significantly lower when the target was presented alone than when it was flanked (aligned: *p* < 0.001; misaligned: *p* < 0.001), yet no differences were found between flanker conditions (*p* = 0.350). For target reporting rate, a significant main effect was found [*F*(2,52) = 34.11, *p* < 0.001, *ƞ*_p_^2^ = 0.57], suggesting that target reporting rate significantly differed between conditions. Here, pairwise comparisons revealed significant differences between the three conditions: the probability of reporting the target orientation when the target was presented alone was significantly higher than when presented with flankers (aligned: *p* < 0.001; misaligned: *p* < 0.001). In addition, the probability of reporting the target when the flankers were aligned was significantly higher compared to the condition where the flankers were misaligned (*p* = 0.012).

**Figure 4:**
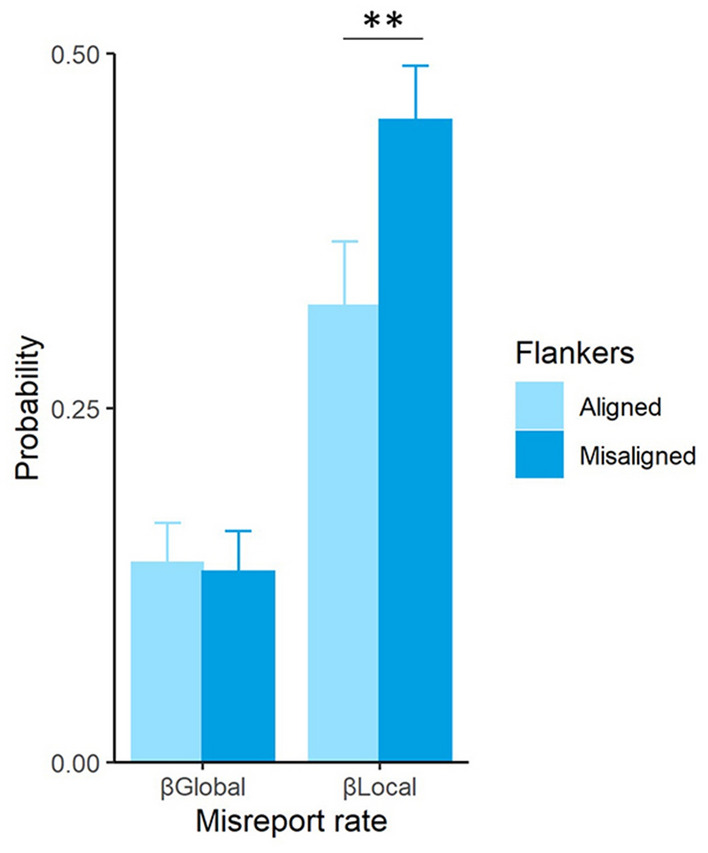
Probability of misreporting the global shape orientation (β_global_) or a local flanker orientation (β_local_) as the target for flankers aligned and flankers misaligned conditions.

Finally, we analyzed the misreport rates to the global and local orientations. Figure 4 depicts the probability of misreporting the global (i.e., either the illusory rectangle or the perceived global shape of the four flankers) or one of the local (i.e., one of the flanker’s) orientation in the two flanker conditions.

The probability of misreporting either a global or a local orientation was significantly different from 0 in both flanker conditions, as assessed by independent *t*-comparisons (all *p*s < 0.001), showing that participants misreported both the global and the local orientations as target orientation in the two flanker conditions. In addition, results revealed no differences in the probability of misreporting the global orientation between flanker conditions [*t*(26) = 0.37, *p* = 0.715], but the probability of misreporting a local flanker orientation was significantly higher when the flankers were misaligned [*t*(26) = −2.94, *p* = 0.007, Cohen’s *d* = −0.56]. As a control, we explored the probability of misreporting the perpendicular (+ 90°) global orientation, which should have a value of 0 since this is the most distant orientation from the actual orientation of the global shape. Indeed, independent *t*-comparisons showed that misreporting the perpendicular global orientation was close to 0 in each flanker condition, mean misreport rate of the global perpendicular orientation being 0.002 and 0.009 in the flankers aligned and flankers misaligned, respectively.

## Discussion

In the present study, we investigated the processing level at which crowding occurs by using crowded displays in which the flankers (local shapes) could form a global configuration (with or without illusory contours) and exploring the extent to which crowding errors depend on these global–local levels. Our results show that observers often misreported the orientation of either the local shapes or the global configuration as the orientation of the local target, suggesting that crowding operates at various levels of visual processing.

The analyses of the model parameters showed a common pattern of significant differences for uncrowded vs. crowded displays^23–27^: Observers reported the target orientation more often (higher target report rate) and more accurately (higher precision) when the target was uncrowded than when it was surrounded by flankers (see Fig. 2); also, the error variability and guessing rate were significantly lower in the uncrowded condition (see Fig. 3). In addition, model fitting for the flanker conditions produced interesting results. While precision performances were similar in both the flankers aligned and flankers misaligned conditions (Fig. 2), the probability of reporting the target when the flankers were aligned was significantly higher compared to the flankers misaligned condition. Note that precision refers to a general index of the error distribution, which indicates how much reports deviated (or not) from the target. The absence of significant differences between the flankers aligned and flankers misaligned conditions shows that observers’ errors were similarly distributed through the 180° response space. The model fitting, on the other hand, decomposes the error distribution into its different components, showing that the alignment of the flankers reduced misreporting a local (flanker) orientation. This reduction in the rate of misreporting a local flanker is what produced a higher Target Response Rate (*P*_*T*_) in the flankers aligned condition (see Fig. 3). In sum, observers’ errors were similarly distributed through the 180° response space as shown by precision indexes in flankers aligned and misaligned conditions, but they misreported individual flankers more (less) frequently in the flankers misaligned (aligned) condition which produced a lower (higher) Target Response Rate in the flankers misaligned (aligned) condition.

Importantly, we analyzed the misreport rates of the global and local orientations (Fig. 4). If crowding occurs at a lower level of visual processing, we expected observers to misreport the orientation of a flanker (local orientation), but not the orientation of the global configuration (illusory rectangle or rectangular configuration) formed by the flankers (global orientation), as the target orientation. Yet, if crowding occurs at a higher level of visual processing, we expected observers to misreport local as well as global orientations as the target orientation. The latter case is what we found. Model comparison showed that a model with both global and local misreports produced a better fit than a model with only local misreports. Furthermore, model fitting revealed that observers significantly misreported both the global and local orientations as target orientation, showing that the global orientation of the flanker configuration is preserved in crowded displays, and suggesting that crowding may occur also at a higher level of visual processing between global and local stimulus representations.

The manipulation of the alignment of the flankers provided some insights into the effect of grouping on the pattern of crowding errors. Even though the global orientation was equally misreported when the flankers were either aligned (illusory rectangle) or misaligned (rectangular configuration), the probability of misreporting a local flanker orientation was significantly higher when the flankers were misaligned, which produced a lower target reporting rate (*P*_*T*_). This suggests that either the perception of the illusory shape or the collinear alignment of the four flankers modulated crowding effects to some extent, in line with previous findings showing that flankers might lose their crowding strength when becoming part of rectangles or good Gestalts^22^. Note that this modulation, however, is not manifested by a higher probability of misreporting the orientation of the illusory rectangle, but rather, by a lower probability of misreporting flanker orientations as target orientation. The contemporary view of grouping is that grouping principles operate at multiple levels, possibly with feedback from higher levels to lower ones^44^. Furthermore, it is commonly accepted that recurrent processing between higher and lower visual areas is required to perceive an illusory form^45,46^ (but see^47^). Doerig, Schmittwilken, Sayim, Manassi and Herzog^48^ recently suggest that recurrent interactions maximize the agreement between different levels of visual processing, such that when flankers are part of a different group than the target, the target is better represented. Presumably then, there is a stronger grouping of the flankers into a separate entity in the flankers aligned than in the flankers misaligned condition, which could lead to a better (i.e., more isolated) representation of the target. A future experiment in which the flankers may form (or not) an illusory shape with the target might support our interpretation of the current pattern of misreport errors, predicting stronger crowding when target groups with the flankers.

The finding that both the global orientation of the rectangular configuration in the flankers misaligned condition and the orientation of the illusory rectangle in the flankers aligned condition produced similar misreport rates might be explained by recent evidence related to the processing of illusory shapes under restrictive visual conditions or when presented in peripheral vision. First, disentangling the perception of the illusory shape from the grouping of the local (flanker) elements is not easy when these configurations are presented under challenging visual conditions. This issue was explicitly studied by Jimenez and Montoro^49^, who displayed masked *illusory* (black “pacmen” and semicircles arranged in such a way that they produced horizontal or vertical illusory bars) and *grouping* (the same pacmen and semicircles rotated in a way that they did not produce illusory figures) primes that could be congruent or incongruent in their orientation with subsequent probe stimuli (vertical vs. horizontal bars). The authors found significant priming effects for *illusory* and *grouping* primes at different prime durations but, crucially, the magnitude of the priming effect was equal for the illusory and grouped primes. Thus, the dissociation of the grouped percept from that generated by the illusory shape is not accomplished easily under restrictive visual conditions. Second, it seems that retinal eccentricity plays a crucial role in illusory shape processing. Bakar, Liu, Conci, Elliott and Ioannides^50^ investigated this by presenting illusory shapes at central and peripheral visual field locations. Their behavioral results revealed that central stimulus presentations elicited faster responses than those presented at one of the four quadrants. In addition, magnetoencephalographic responses to illusory figures showed that for central presentations, specific responses to illusory figures peaked first in V1/V2 (96–101 ms), and then in the lateral occipital complex (LOC; 132–141 ms). For peripheral presentations, the relative modulation towards illusory figures was markedly reduced in V1/V2 and LOC while prominent activation peaks shifted to the fusiform gyrus (from 200 ms onwards). Thus, the processing of illusory shapes in the periphery required longer latencies and the involvement of higher-level visual areas. Overall, this combined evidence might explain the absence of differences in misreporting the global orientation between flanker conditions observed in our results. Additionally, in a very small number of trials, the random rotation of the flankers might produce some weaker illusory contours or partial illusory shapes in the flanker misaligned condition of the same orientation of the global configuration. This might also help to understand the absence of significant differences in reporting the global orientation between flanker conditions.

While previous research on the effect of grouping processes in crowding have thoroughly explored how crowding strength varies when flankers group together or are perceptually separated from the target, here we introduced a novel experimental design to evaluate the pattern of misreport errors when targets are presented within perceptually grouped configurations. The findings presented in this study integrate with recent evidence^22,37,51^ showing that the global stimulus configuration is preserved in crowding displays and plays a crucial role in crowding, stressing the necessity of including grouping-like processes as a fundamental explanation of crowding. We consider these results difficult to be explained by an early and single pooling stage. Instead, our finding can be explained by models that comprise both bottom-up and top-down processing that enable observers to perceptually group independent elements of a scene^36^. It has been recently proposed that global grouping, segmentation processes and top-down processing are crucial in explaining how the brain deals with global configurations in crowding^37,48,51^. Therefore, crowding models which incorporate recurrent processing^36,48^, in contrast with explanations based solely on local and feedforward computations, might produce better explanations for grouping and configurational effects in crowding.

In sum, the present results suggest that crowding occurs simultaneously across multiple levels of visual processing, both at a lower level between local shapes but also at a higher level between global and local shapes. Since both local flanker information and global shape representations are preserved (not averaged) in crowded displays, our results provide a further challenge to low-level “pooling” or averaging models of crowding. The development of new pooling models^5,52^, which incorporate the possibility of multilevel crowding and account for complex target-flanker interactions, might lead to a better explanation of the phenomena of crowding in general, and the current results in particular.

## Data Availability

The methods used and the data analyzed in the present study are available in the Open Science Framework (OSF) repository, in the following link: https://osf.io/f3cz8/?view_only=b47af6157a1941d48edbddd3b0ecea10.
